# Mussel-Inspired Catechol-Functionalized Hydrogels and Their Medical Applications

**DOI:** 10.3390/molecules24142586

**Published:** 2019-07-16

**Authors:** Wei-Yan Quan, Zhang Hu, Hua-Zhong Liu, Qian-Qian Ouyang, Dong-Ying Zhang, Si-Dong Li, Pu-Wang Li, Zi-Ming Yang

**Affiliations:** 1 Department of Applied Chemistry, School of Chemistry and Environmental Science, Guangdong Ocean University, Zhanjiang 524088, China; 2 Key Laboratory of Tropical Crop Products Processing of Ministry of Agriculture and Rural Affairs, Agricultural Product Processing Research Institute, Chinese Academy of Tropical Agricultural Sciences, Zhanjiang 524001, China

**Keywords:** mussel adhesive protein, catechol, hydrogel, medical application

## Abstract

Mussel adhesive proteins (MAPs) have a unique ability to firmly adhere to different surfaces in aqueous environments via the special amino acid, 3,4-dihydroxyphenylalanine (DOPA). The catechol groups in DOPA are a key group for adhesive proteins, which is highly informative for the biomedical domain. By simulating MAPs, medical products can be developed for tissue adhesion, drug delivery, and wound healing. Hydrogel is a common formulation that is highly adaptable to numerous medical applications. Based on a discussion of the adhesion mechanism of MAPs, this paper reviews the formation and adhesion mechanism of catechol-functionalized hydrogels, types of hydrogels and main factors affecting adhesion, and medical applications of hydrogels, and future the development of catechol-functionalized hydrogels.

## 1. Introduction

Humans are always inspired by organisms with unique capabilities in nature. A good example is marine mussels, they can firmly adhere to different surfaces in the seawater by secreting mussel adhesive proteins (MAPs) through the byssus. Research has been conducted for main components of MAPs, six *Mytilus edulis* foot proteins (Mefps) including Mefp-1, Mefp-2, Mefp-3, Mefp-4, Mefp-5, and Mefp-6 have been identified as the main components of MAPs. Of them, Mefp-3, Mefp-5, and Mefp-6 play an important role in adhesion. Notably, all three contain 3,4-dihydroxyphenylalanine (DOPA) with respective molar percentages of 20–25% [1], ~30% [2], and ~3% [3]. Recently, another fifteen new proteins in *Mytilus Californianus* foot proteins (Mcfp) such as Mcfp-7p v1, Mcfp-8p, Mcfp-7p v2, Mcfp-9p v1, Mcfp-10p, Mcfp-9p v2, Mcfp-11p, Mcfp-12p, Mcfp-13p, Mcfp-14p, Mcfp-15p, Mcfp-16p, Mcfp-17p, Mcfp-18p, and Mcfp-19p have been tested out [4]. Further research revealed that DOPA was the key factor of interaction between the material and the surface [5]. The catechol groups in the molecule can adhere to different surfaces in wet environments, including silica [6,7,8], metal ions [9,10], metal oxides [11,12], biological tissues [13,14,15], and especially the mucosa [13,16], through physical and chemical processes. For this reason, extensive research has focused on the catechol group.

Hydrogels with 3-dimensional (3D) polymeric networks are a common formulation with wide applications due to their properties such as soft texture, shape preservation, and high water-absorbing capacity. Since the catechol group is highly adhesive to biological tissues, hydrogels prepared from catechol-functionalized materials are medically useful [17] for biological tissue adhesion [18,19,20], sustained drug release [21,22,23,24], coatings for biological and clinical devices [25,26,27,28,29], and tissue engineering [30,31,32,33].

There are many reviews on catechol-functionalized materials [34,35,36,37], especially on MAPs [38,39,40,41,42,43,44]. Many of them mainly summarized the adhesive mechanisms of MAPs which were as bioadhesive materials in wet environments in addition to their biomimetic applications in various fields. Seldom of them concerned the catechol-functionalized hydrogels which were applied in medicine. Based on a discussion of the mechanism of MAPs, this study describes the formation and adhesion mechanisms of catechol-functionalized hydrogels, hydrogel preparations and types, and the main factors affecting adhesion. We also review the medical applications of catechol-functionalized hydrogels, such as tissue adhesion, biomedical coatings, and drug delivery systems.

## 2. Mussel Adhesive Proteins

**Figure 1:**
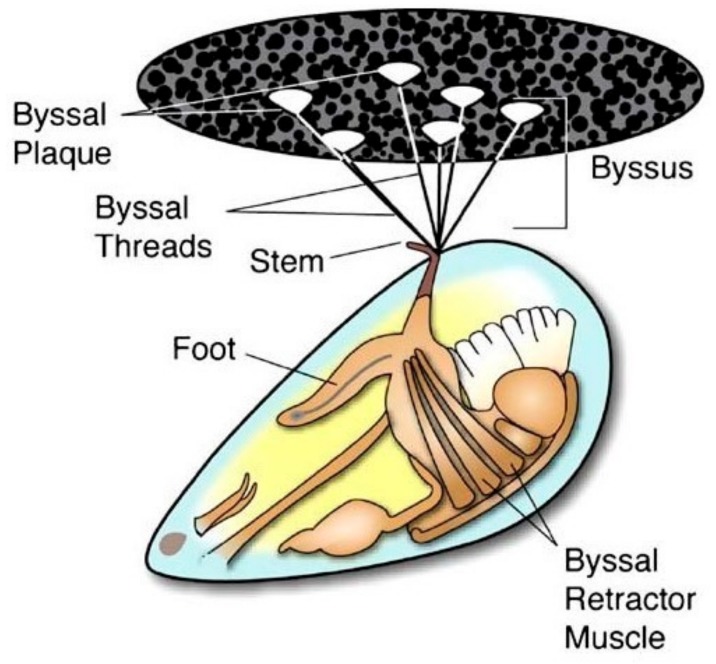
Anatomy of *Mytilus edulis* mussel and byssus structures. Reproduced with permission from Ref. [39], Copyright 2007 Springer Science + Business Media, LLC.

Mussels are well-known marine organisms that can firmly adhere to the surface of rocks, ships, and others by secreting MAPs. As shown in Figure 1, the mussel is connected to the external environment through byssal threads and plaques, both of which are secreted from foot gland. These mussel secretions mainly consist of polyphenolic proteins, collagen, and polyphenol oxidase [39], the arrangement of which provides excellent water resistance and high strength [45]. Mussel adhesion strength and efficiency are also affected by environmental factors such as temperature, salinity, pH, substrate, and season, as well as the organism’s metabolic status [46].

**Figure 2:**
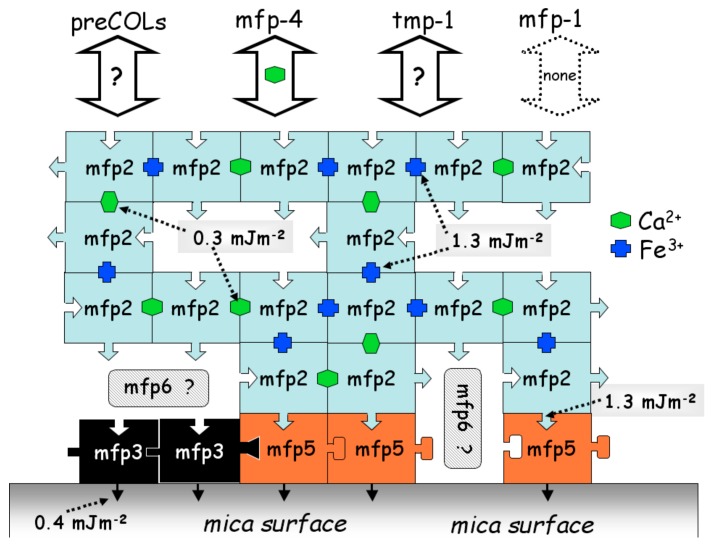
Interactions between metals and proteins in the byssal plaque of Mytilus. Reproduced with permission from Ref. [51], Copyright 2004 Wiley-VCH Verlag GmbH & Co. KGaA.

Understanding MAPs adhesion mechanisms can contribute to their development and utilization. MAPs effectively adhere to both organic and inorganic surfaces. However, the mechanism depends on many factors and it is not fully understood. It has been reported that water-soluble peptides containing lysine residues play key role in effective adhesives [47]. It is widely accepted that the main reasons underlying MAPs adhesion are the high proportion of post-translationally modified amino acids such as DOPA [48], and the redox status of catechol groups in the DOPA side groups [49]. Indeed, interaction of metal ions (e.g., Fe^3+^, Cu^2+^, Ca^2+^ and Ti^3+^) with proteins [9,50,51,52] also play a significant role in the cohesion and adhesion process of MAPs. Hwang et al. [51] investigated the interaction between Mefps depending on calcium and iron. As shown in the Figure 2, mussel foot protein 2 (i.e., Mfp-2) interacts directly with Mfp-5 and with itself in the presence of calcium or iron. Mfp-2 can also interact with Mefp-4 through calcium, but has no interaction with Mfp-1.

Generally, two simultaneous procedures, which are cohesion among proteins and adhesion of proteins on substrate, have been revealed in MAPs adhesion mechanism. MAPs contained DOPA have achieved covalent self-crosslinks between MAPs and biological substrate during transforming catechol groups into quinones by polyphenol oxidase enzyme in mussels.

**Figure 3:**
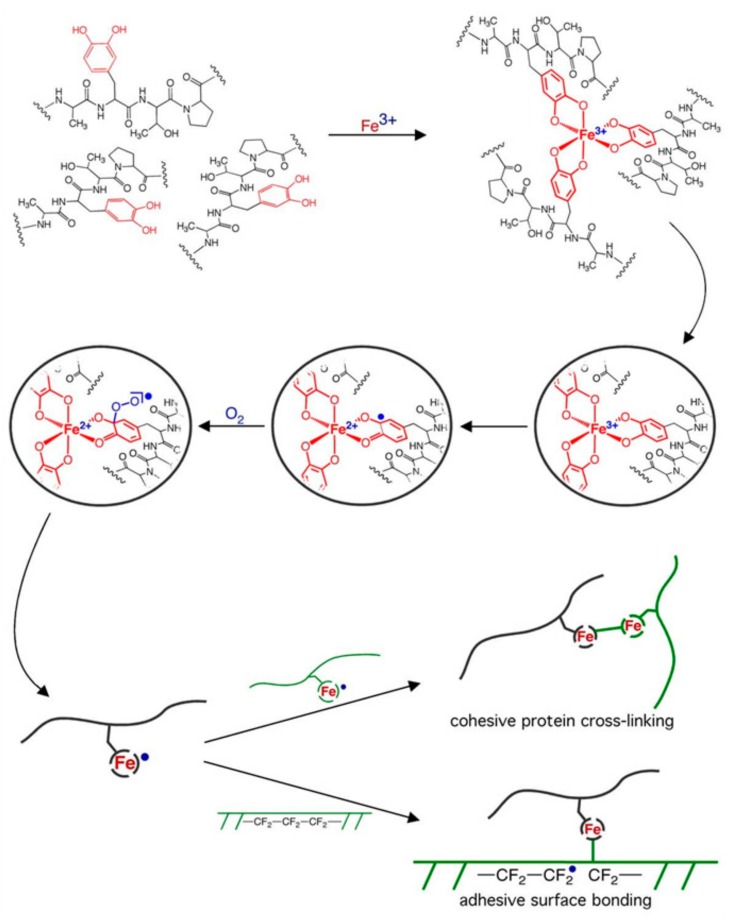
Adhesion mechanism of iron-induced 3,4-dihydroxyphenylalanine (DOPA). Reproduced with permission from Ref. [53], Copyright 2009, Elsevier Ltd.

Wilker proposed an iron-induced DOPA adhesion mechanism for MAPs [53]. As shown in Figure 3, when the DOPA in Mefp-3 and Mefp-5 proteins are mixed with Fe^3+^ in aqueous environments, the side-chain catechol groups of DOPA can be chelated with Fe^3+^ to aggregate the three protein chains into a Fe(DOPA-protein)_3_ complex. Under the influence of catechol dioxygenase [54], the Fe^3+^ in the complex undergoes valence tautomerization and forms a Fe^2+^ semiquinone complex under redox equilibrium conditions. The semiquinone complex reacts with O_2_ to form a free radical that can exist or be converted to other free radicals. The free radical-containing proteins can undergo free-radical coupling to provide cohesive bulk cross-linking. The free radicals can also be attached to the substrate surface by covalent bonds. Wilker suggested that although this iron-induced adhesion mechanism was consistent with most research data, many details remained unclear [55]. Multiple mechanisms [56,57] or other amino acids [58] may be involved.

## 3. Formation Mechanisms of Catechol-Based Hydrogels

**Figure 4:**
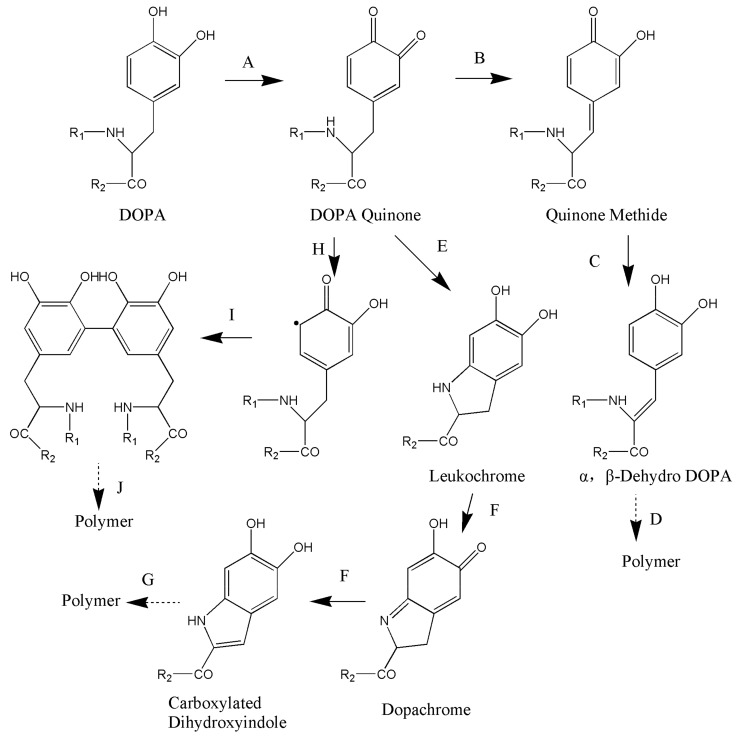
Oxidative Reactions and Possible Cross-Linking Pathways of DOPA. (**A**) Oxidation by catechol oxidase or other oxidizing reagents. (**B**) Tautomerization of DOPA quinone. (**C**) Release of an α proton. (**D**) Cross-linking via a pathway similar to that occurring in insect cuticle sclerotization. (**E**) Internal cyclization with R_1_ = H. (**F**) Rearrangement of cyclized DOPA derivatives. (**G**) Cross-linking by a pathway resembling melanin formation. (**H**) Aryloxy free radical generation. (**I**) Phenol coupling. (**J**) Further oxidation to form a cross-linked polymer. Dashed arrows indicate poorly understood pathways that lead to cross-linked polymer formation. Reproduced with permission from Ref. [59], Copyright 2002, American Chemical Society.

Similar to MAPs adhesion, various redox states of catechol and its derivatives result in the formation of catechol-based hydrogels. Figure 4 shows three reaction pathways involved in catechol-based hydrogel formation [59]. The formation of hydrogels may be the result of one or more forms of cross-linking.

First, cross-linking can occur in a way similar to that occurring in insect cuticle sclerotization. As shown in A→B→C→D in Figure 4, DOPA is oxidized to semiquinone and quinone in the presence of chemical oxidants (e.g., O_2_, NaIO_4_, and H_2_O_2_) and peroxidases (e.g., tyrosinase and horseradish peroxidase) (Figure 4A) [60]. The catechol group of this reactive quinone structure forms a quinone methide by tautomerization (Figure 4B). Next, α, β-dehydro-DOPA is formed by the release of α protons by non-enzymatic action (Figure 4C) [61], resulting in dimer and polymer formation [59]. Finally, a polymer is produced (Figure 4D). The formation of quinone methide and α,β-dehydro-DOPA may be one of the cross-linking pathways [59].

Cross-linking can also occur in a way resembling melanin formation in mammals. As shown in A→E→F→G in Figure 4, DOPA is first oxidized to quinone (Figure 4A) and then rapidly converted to leukochrome by intramolecular cyclization (Figure 4E) in the presence of chemical oxidants (e.g., O_2_, NaIO_4_, and H_2_O_2_) and peroxidases (e.g., tyrosinase and horseradish peroxidase). Finally, these cyclized products and unoxidized catechol groups are polymerized to melanin (Figure 4G) [34].

Lastly, cross-linking can occur in the form of benzene–benzene cross-linking [62] As shown in A→H→I→J in Figure 4, DOPA is oxidized to quinone in the presence of chemical oxidants (e.g., O_2_, NaIO_4_, and H_2_O_2_) and peroxidases (e.g., tyrosinase and horseradish peroxidase) (Figure 4A). This reactive quinone structure can lead to the generation of aromatic oxidative radicals (Figure 4H) that cross-link with the adjacent catechol group to form dimers (Figure 4I) and subsequently polymers (Figure 4J) [63]. Sánchez-Cortés et al. [64] performed surface-enhanced Raman spectroscopy to show that the catechol group could initiate polymerization by C-C and C-O-C ether linkages at neutral pH. The benzene ring can also form cation-π non-covalent interactions with a surface with cations [65,66].

## 4. Types of Catechol-Based Hydrogels

Catechol-based hydrogels are commonly prepared by oxidant-induced cross-linking [67,68,69]. The hydroxyl groups in hydrogel of containing catechol groups can easily give up electrons and be oxidized under the influence of oxidizing agents (e.g., oxygen, hydrogen peroxide, and periodic acid) and enzymes (e.g., tyrosinase and horseradish peroxidase). The oxidized catechol groups have high chemical activity and can be grafted onto the molecular chains of other polymers to form a gel through oxidative cross-linking. Based on polymer functionality and the use of other substances that endow hydrogel with unique properties, catechol-based hydrogels can be classified into non-functional, nanocomposite, thermosensitive, and pH responsive hydrogels.

### 4.1. Non-Functional Hydrogels

**Figure 5:**
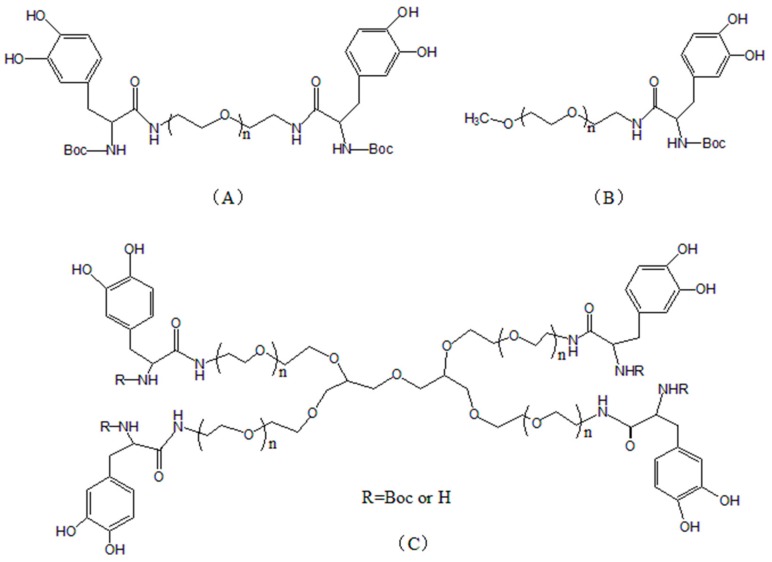
Chemical structures of DOPA-modified PEGs. (**A**) DOPA-modified PEGs containing two arms. (**B**) DOPA-modified PEGs containing one arm. (**C**) DOPA-modified PEGs four arms. Reproduced with permission from Ref. [59], Copyright 2002, American Chemical Society.

Non-functional hydrogels are prepared by the method of modification of natural or synthetic polymers with DOPA and its derivatives. These hydrogels do not possess the special properties such as thermosensitive or pH-responsive yet they have normal adhesive capacity. They are an important hydrogel type because of their high adhesive strength and easy preparation. As a non-toxic, non-irritating, water-soluble, and biologically compatible polymer, polyethylene glycol (PEG) has been widely used in the medical field. Potential application such as engineering and drug release may be found by combination of PEG and DOPA using standard carbodiimide coupling chemistry method. In a study by Lee et al. [59], DOPA was grafted onto linear and branched PEGs, as shown in Figure 5A–C. Hydrogels were then prepared by oxidation of sodium metaperiodate, horseradish peroxidase, and tyrosinase. They found that PEG-DOPA end groups containing more DOPA could polymerize into a network structure to rapidly form a gel (Figure 5C) in less than 1 minute. UV-Vis spectroscopy revealed that oxidation of the catechol group side chain in PEG-DOPA resulted in the formation of highly reactive dopaquinone, which further reacted to form cross-linked products via one of several pathways, depending on whether the amino group of PEG-DOPA was protected. N-Boc protected PEG-DOPA was cross-linked via phenol coupling and quinone methide tanning, whereas PEG-DOPA containing a free amino group was cross-linked via a pathway that resembled melanogenesis. Hydrogels with desired properties can be formulated via different cross-linking mechanisms by controlling the chemical structure of the DOPA, the PEG backbone, and the cross-linking reagent concentration. Gelation time was highly dependent on the polymer structure, as well as the type and concentration of oxidizing reagent [70]. The higher the enzyme activity and concentration, the faster the reaction. The shortest gelation time was found at a 1:1 molar ratio of chemical oxidant (NaIO_4_) to DOPA.

**Figure 6:**
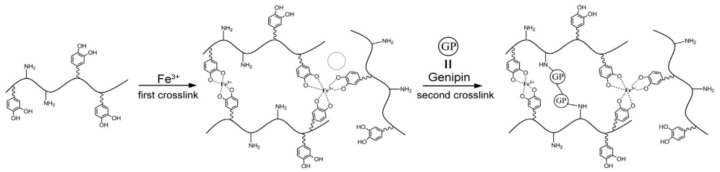
Double cross-linking and formation of hydrogels. Reproduced with permission from Ref. [71], Copyright 2016, Acta Materialia Inc.

The oxidative cross-linking of hydrogels consumed catechol groups, which resulted in the poor strength of hydrogel and adhesive strength when the small amount of DOPA was grafted to polymers. Moreover, cross-linking strength is positively correlated with catechol group content. To achieve biocompatible, rapid, and strong adhesion to wet tissues, Fan et al. [71] designed a double cross-linked tissue adhesive hydrogel (DCTA). As shown in Figure 6, DOPA was grafted onto a gelatin backbone by carbodiimide coupling chemistry, so that the catechol group could perform strong wet adhesion on tissue surfaces. Then, Fe^3+^ was added to form a catechol-Fe^3+^ complex, which was unstable due to non-covalent binding. In order to enhance the crosslinking strength, genipin, which is a hydrolyzed product of geniposide by β-D-glucosidase and it is an excellent natural biological crosslinking agent, was used to cross-link the gelatin molecules. Genipin can react with primary amino groups in polymers, such as gelatin, chitosan, and polylysine, to form stable cross-linking products, which endows DCTA with a double-cross-link adhesion mechanism. Without any purification, fresh porcine skin and articular cartilage was chosen as adherends to closely mimic clinical conditions. The adhesion strength of DCTA on the fat layer (inside) and the wet collagen layer (outside) of each wet porcine skin and cartilages were tested and the results are shown on Table 1. The highest adhesion strength on cartilages was up to 194.4 ± 20.7 kPa, which presents a highly promising product as a biological glue for internal medical use including internal tissue adhesion and sealing.

**Figure 7:**
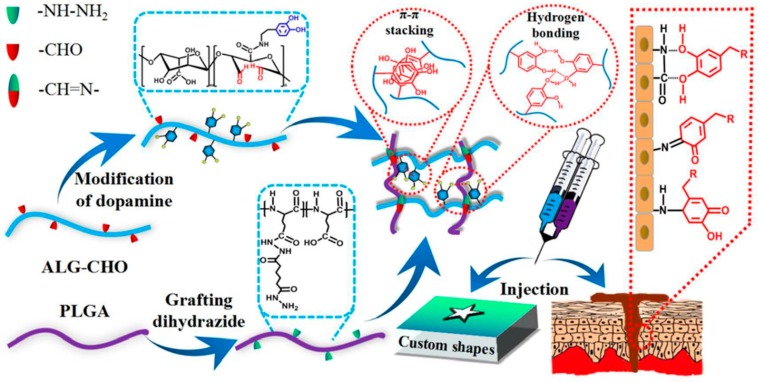
Schematic illustration of the mussel-inspired PLGA/ALG-CHO-catechol adhesive injectable hydrogels. The combination of aldehyde-modified alginate backbones (ALG-CHO)-catechol and polyglutamic acid with hydrazide (PLGA-ADH) led to self-cross-linking of a three-dimensional (3D) network in the form of hydrogels, allowing custom shapes to be completely matched and filled. The hydrogels with abundant free catechol groups were inspired by marine mussels, forming various interactions like π-π stacking and hydrogen bonds in the 3D network, and adhering to different surfaces even in wet environments. Reproduced with permission from Ref. [72], Copyright 2018, Royal Society of Chemistry.

Yan et al. [72] described a method for preparing mussel-inspired injectable hydrogels through a Schiff base reaction. As shown in Figure 7, hydrogels were prepared by double cross-linking via the Schiff base reaction and oxidative cross-linking of catechol groups was achieved by grafting DOPA onto the aldehyde-modified alginate backbones (ALG-CHO) and modifying polyglutamic acid with hydrazide (PLGA-ADH). The cross-linking of hydrogels and 3D network formation were based on the Schiff base reaction to avoid introducing small molecule oxidants and maintain the chemical structures of the catechol-functional groups. The catechol group endowed the injectable hydrogels with good mechanical strength, robust adhesion and strong self-healing ability due to the π-π stacking between the hydrogen bond and catechol group.

### 4.2. Nanocomposite Hydrogels

**Figure 8:**
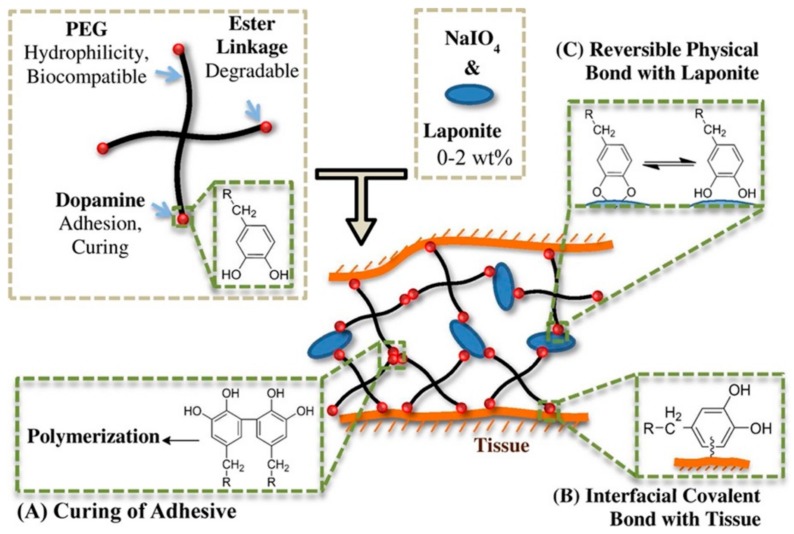
Preparation of nanocomposite hydrogel and proposed three types of cross-links in this system. (**A**) Curing of adhesive. (**B**) Interfacial covalent bond with tissue. (**C**) Reversible physical bond with laponite. Reproduced with permission from Ref. [73], Copyright 2014, American Chemical Society.

Non-functional catechol-functionalization hydrogels always have weak crosslinking and low mechanical strength, limiting the application in tissue engineering scaffolds of medicine. As we know, materials can acquire special properties when the particle size is reduced to a nanoscale of approximately 1 to 100 nm. The addition of nanoparticles to catechol-based hydrogels can not only enhance the crosslinking density and thus elevated cohesion of hydrogels, but also impart various properties (e.g., antimicrobial and strong adhesive strength) due to the nano effect. According to Liu et al. [73], who prepared the injectable nanocomposite hydrogels, as shown in Figure 8, the nanosilicate Laponite was mixed with the oxidant sodium metaperiodate (NaIO_4_) and placed into a DOPA-modified 4-arm poly (ethylene glycol) (PEG-D4) solution. The catechol groups of DOPA reacted to form a hydrogel in the oxidation of sodium metaperiodate. Due to the presence of nanosilicate, they suggested that the hydrogel system involved three processes of cross-links during the oxidation of DOPA by sodium metaperiodate. As shown in Figure 8, DOPA was first oxidized to highly reactive quinone, indicating cross-linking via aryloxy coupling (Figure 8A) [59]. Second, quinone reacted with the reactive groups in the biological tissue (e.g., -NH_2_ and –SH) to form strong, irreversible covalent bonds (Figure 8B) [14,57]. Finally, a strong hydrogen bond (Figure 8C) was formed between the hydroxyl groups of catechol moieties and Laponite [74], thereby reinforcing the hydrogel’s network structure. The time required for hydrogel formation was affected by the NaIO_4_/DOPA molar ratio and Laponite. The curing time was 1.81 ± 0.12 min at a 0.5 molar ratio of NaIO_4_ to DOPA when Laponite was not added. The curing time was significantly reduced with the addition of Laponite. For example, it decreased to 0.92 ± 0.03 and 0.33 ± 0.06 min, respectively, when 1 and 2 wt % Laponite was added at a 0.5 molar ratio of NaIO_4_ to DOPA. Liu et al. [75] also developed a novel moldable nanocomposite hydrogel which transitioned from a reversibly crosslinked network formed from dopamine-Laponite interfacial interactions to a covalently crosslinked network through the slow autoxidation and crosslinking of catechol moieties. The advantages of the nanocomposite hydrogel are mouldability and without using cytotoxic oxidants, so that it can apply in sealing tissues with non-flat geometries, such as a sutured anastomosis.

However, biocompatibility and degradability of Laponite based nanocomposite hydrogels blocks their application in tissue adhesion. Chitin nanocrystals, which mainly come from shrimp and crab shells, are natural polysaccharides with histocompatibility and absorbability. Xu et al. [76] prepared a nanocomposite adhesive hydrogel by dispersing chitin nanocrystals (ChiNCs) into a DOPA-grafted citrate-based tissue adhesive. They found that ChiNCs were finely dispersed in the hydrogel matrix and endowed the adhesive with extra cross-links to enhance cohesion performance. The lap shear adhesion strength of the hydrogel significantly increased with higher ChiNCs content. Moreover, the addition of ChiNCs imparted a low swelling ratio, good cytocompatibility, and superior applicability in soft tissue adhesion to the hydrogel.

In practical applications, incomplete sterilization of hydrogels or inadequate disinfection of tissue results in the failure adhesive of hydrogels to possible infection related to the secretion from microorganisms. As an antimicrobial, nano-silver has a long medical application history. It can be applied to medical hydrogel adhesives to provide an antimicrobial property. In a study by Fullenkamp et al. [19], silver ions were used to oxidize catechol groups, leading to hydrogel formation from catechol-functionalized PEG, while silver ions were reduced to nano-silver particles during oxidation. Silver release was sustained for 2 weeks in phosphate buffer solution, thus achieving both adhesive and antimicrobial properties. The hydrogel was also found to resist bacterial and mammalian cell attachment, consistent with the antifouling properties of PEG.

### 4.3. Thermosensitive Hydrogels

**Figure 9:**
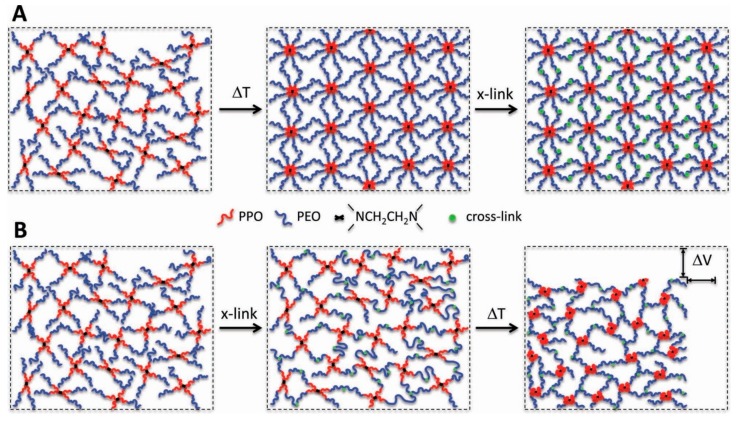
Schematic illustration of chemically cross-linked poly(propylene oxide)-poly(ethylene oxide) (PPO-PEO) block copolymer thermoresponsive gels (not to scale), demonstrating the significance of controlling the sequence of thermal transition and chemical cross-linking. (**A**) In the “tandem” method of Cellesi et al. [82,83], chemical cross-linking occurs after thermal equilibration. (**B**) In the present method, chemical cross-linking of cT is intended to occur prior to thermal equilibration, producing a PPO-PEO block copolymer network. Subsequent warming induces thermal transition of the PPO segments from hydrophilic to hydrophobic, producing volumetric shrinkage and toughening the network. Reproduced with permission from Ref. [81], Copyright 2013, Wiley-VCH Verlag Gmbh & Co. KGaA.

Thermosensitive hydrogels are polymer hydrogels that change volume with temperature and exhibit lower critical solution temperature (LCST) phase behavior. Most synthetic polymer hydrogel as tissue adhesives and sealants swell in physiological conditions, which can result in mechanical weakening and adverse medical complications. The use of thermosensitive polymers may be able to prevent the swelling of polymer hydrogel tissue adhesives [77,78,79]. Barrett et al. [80] developed a thermosensitive hydrogel cross-linking system to prepare zero- or negative-swelling thermosensitive hydrogels as surgical adhesives based on catechol-modified poly(propylene oxide) (PPO)- poly(ethylene oxide) (PEO) block copolymers using the chemical cross-linking of catechol group and the subsequent thermal transformation of PPO. As shown in Figure 9B, the mixture of containing catechol groups polymer and chemical oxidant will rapidly cross-link to form a hydrogel, which meets the chemical cross-linking requirements for hydrogel formation. After implantation, PPO will shift from a hydrophilic to a hydrophobic state with increasing temperatures, and physical agglomeration occurs again, thereby leading to volumetric reduction and mechanical toughening [81]. This approach can be easily adapted for other thermosensitive block copolymers and cross-linking strategies, representing a general approach for controlling swelling and enhancing mechanical properties of polymer hydrogels used in medical contexts.

**Figure 10:**
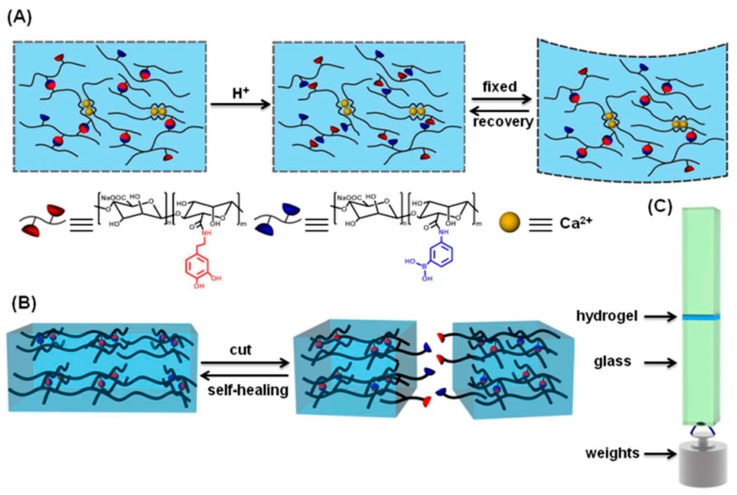
Schematic illustration of (**A**) shape memory, (**B**) self-healing, and (**C**) adhesion properties of phenylboronic acid-dopamine-grafted (PBA-DA) hydrogels. Reproduced with permission from Ref. [89], Copyright 2016, Royal Society Chemistry.

Poly(N-isopropylacrylamide) (PNIPAM) hydrogels are widely studied thermosensitive hydrogels with potential medical applications due to their LCST (32 °C) being close to human body temperature. Li et al. [84] prepared an injectable thermosensitive hydrogel (DNODN) (Figure 10A) with catechol-functionalized PNIPAM as the thermosensitive A block and poly(ethylene oxide) (PEO) as the hydrophilic B block using hydrogen bonding between catechol groups and aromatic interactions. The hydrogel prepared through self-assembly of this triblock copolymer was demonstrated to undergo rapid thermoresponsive sol-gel transitions and heal spontaneously from repeated damage. In addition, the DNODN hydrogel could prevent non-specific cell attachment due to the strong antifouling properties of PEO. It is noteworthy that the A block, catechol-functionalized PNIPAM, provided a hydrophobic microenvironment that effectively retarded the oxidation of catechol groups.

### 4.4. pH-Responsive Hydrogels

Multi-pH-responsive design will facilitate control over the mechanical properties of the hydrogels and that reversible nature of the complex bonds will impart self-healing properties to the materials. The hydrogels will display the maximum mechanical strength at a pH close to the polymer’s pI value, which in this case is close to the pK_a_ value of functional groups. It is well known that phenylboronic acid (PBA) groups are pH-responsive. They can form complexes with cis-diol molecules (e.g., PVA, glucose, and catechol group) when the pH of the aqueous medium is higher than the pKa of the PBA groups (pKa ≈ 8.5) [85,86,87]. Novel supramolecular smart materials can be developed for a range of potential biomedical applications based on this property of PBA [88]. Li et al. [89] proposed a simple but effective strategy to impart versatility to supramolecular hydrogels, which may contribute to the development of functional materials. The authors prepared PBA-modified sodium alginate (Alg-PBA) and dopamine-grafted sodium alginate (Alg-DA) that were then complexed by reversible PBA-catechol bonds formed by PBA and catechol under alkaline conditions to obtain a supramolecular hydrogel (Figure 10A). The dynamic nature of PBA-catechol bonds imparted the hydrogel with self-healing ability and pH-responsive shape-memory properties (Figure 10B). Furthermore, the presence of mussel-involved catechol groups imbued the prepared hydrogel with adhesive properties (Figure 10C).

Narkar et al. [90] prepared pH responsive adhesive hydrogel with catechol-boronate complex which remained reversible and the interfacial binding property of the adhesive that was tuned with changing pH in successive contact cycles. Acrylic acid (AAc), an acidic anionic monomer, was introduced into the adhesive network, and shifted the pH of catechol-boronate complexation to a more basic pH. With increasing AAc contents, hydrogels demonstrated strong adhesion to quartz substrate at a neutral to mildly basic pH (pH 7.5–8.5). With tuning pH to 9.0, the catechol-boronate complex was formed and the adhesion values were significantly reduced (18- and 7-fold reduction compared to values measured at pH 7.5 and 8.5, respectively). However, the formed complex was dissociated when tuned pH to 3.0, and the elevated adhesive property was recovered.

**Figure 11:**
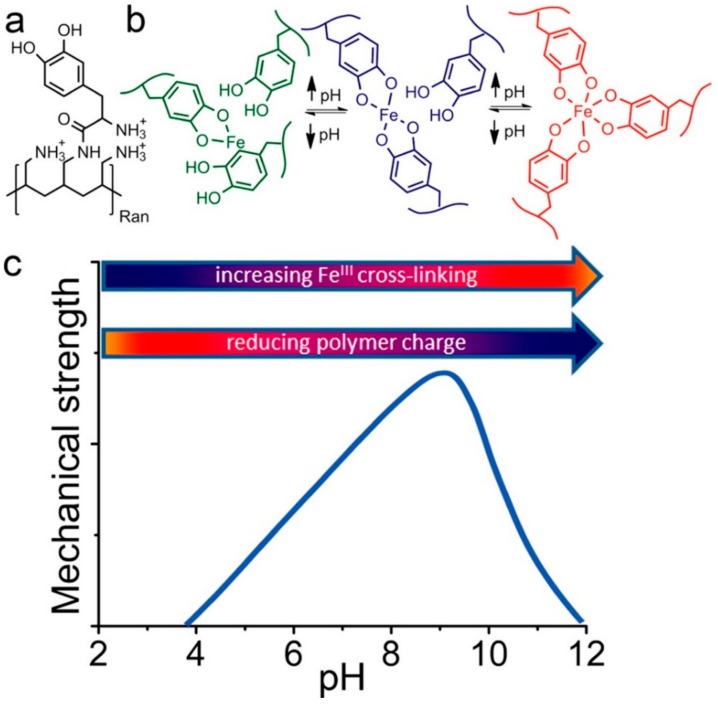
DOPA was randomly grafted onto polyallylamine yielding the polymer shown in (**a**). There are several pH responsive parts of the polymer. The amine side chain itself is involved in an acid/base equilibrium with a pKa ~9.3–9.7 as determined by potentiometric titrations. Furthermore, the catechol groups can be oxidized to the quinone form or cross-linked by FeIII in the pH-dependent manner shown in (**b**). The multi-pH-responsive design is believed to achieve the maximum mechanical strength around the polymer’s pI value as shown in (**c**): As the pH increases from acidic values, the polymer is increasingly cross-linked due to the formation of bis- and tris-(DOPA)Fe complexes. The polymer charge is simultaneously reduced, leading to reduced polymer hydrophilicity and interpolymer chain repulsion. Above a threshold pH value, this leads to gel collapse and reduced strength. Reproduced with permission from Ref. [92], Copyright 2013, American Chemical Society.

More than 75% of DOPA residues in Mefp-5 are next to charged lysine residues [2,91], which may result in changes in the properties of the protein itself, as well as the redox reaction of DOPA and metal coordination. To better simulate MAPs, Krogsgaard et al. [92] proposed a pH-dependent, self-healing, responsive hydrogel system. In their work, DOPA was grafted onto highly positively charged amine-functionalized polyallylamine (Figure 11a) and then reacted with iron to form an iron-coupled multi-responsive system (Figure 11b). The degree of cross-linking was pH controlled through DOPA/iron coordination chemistry. The system rapidly formed a self-healing, high-strength hydrogel when pH was increased. Above the threshold value, the hydrogel collapsed due to reduced repulsion between polymer chains. A biostable gel system was thereby obtained by pH regulation.

Generally, an acidic condition decreases the curing rate and adhesive strength, while an alkaline environment lessens tissue adhesion due to reduced concentration of catechol. The bulk of research on catechol-functionalized hydrogels concerned an alkaline environment, but less studied acidic conditions which correspond to organizational environments. This may be related to the intrinsic properties of catechol.

## 5. Interactions of Catechol-Functionalized Hydrogels on the Surface of Biological Tissues

Due to the presence of a layer of water molecules on the surface of a moist substrate, hydrogels cannot directly contact the substrate surface and thus cannot achieve strong adhesion. MAPs are special that they can adhere strongly to the wet interface through the protein arrangement and the key role of DOPA. The catechol group plays an important role in MAP adhesion, allowing the catechol-functionalized hydrogels to adhere to the soft tissue surface in a wet environment.

Lee et al. [57] investigated the substrate of DOPA and its adhesion to the surfaces of organic materials and inorganic metal oxides associated with oxidation in a single-molecule assay. They found that single-molecule DOPA had a surprisingly strong, reversible non-covalent interaction on the wet metal oxide surface. Oxidized DOPA had a reduced interaction with metal oxides but an enhanced one with organic materials. This was due to the formation of strong, irreversible covalent bonds. Guvendiren et al. [14] examined the adhesion of DOPA-functionalized membranes to a hard substrate and soft tissue. In their experiment, DOPA was incorporated into a diblock copolymer (PS-PEO-Boc-DOPA) and triblock copolymer (PMMA-PMAA-PMMA) to form hydrogels, and then placed in contact with TiO_2_ and pig skin. The results showed that DOPA strongly adhered to the surface of pig skin. The strongest adhesion was obtained when the catechol group was in contact with the tissue surface and oxidized. Peng et al. [93] found that hydrocaffeic acid (HCA)-grafted hydroxymethyl chitosan (HECTS) hydrogels (HCA-g-HECTS) prepared by periodate-induced cross-linking exhibited a stronger storage modulus and greater temperature stability than hydrogels obtained by Fe^3+^-triggered cross-linking; the hydrogels prepared by periodate-induced oxidative cross-linking possessed a high adhesion strength of 73.56 kPa against wet rat skin.

In summary, catechol-based hydrogels adhere to biological tissue surfaces by reacting with the reactive groups (e.g., -NH_2_ and –SH) in biological tissues through oxidative cross-linking of catechol groups to form irreversible covalent bonds. Table 2 shows the adhesive strength of MAPs, recombinant MAPs and catechol-functionalized biomedical hydrogels on different substrates.

### 5.1. Effect of Oxidation State on Hydrogel Adhesion

Hydrogels, adhere to tissue substrates, and are highly dependent on oxidation state of catechol residues which exists predominantly in its reduced form at an acidic pH and exhibits elevated adhesive strength to inorganic substrates [12,58,97]. With an increasing pH it approaches and exceeds the first dissociation constant of the catechol -OH group (pka1 = 9.3), the catechol groups autoxidize to quinone form that results in a reduced adhesive strength to inorganic substrates but are elevated to biological substrates [57,98]. Addition of chemical oxidants (i.e., periodate) irreversibly oxidizes catechol groups benefitting the formation of hydrogels but resulting in reduced adhesive properties. On the other hand, quinone, the product of oxidation, can form interfacial covalent bond with biological substrates by reacting with various nucleophilic functional groups (i.e., -NH_2_, -SH, imidazole), forming an interfacial covalent bond [57]. To prevent oxidation or preserve the reduced state of catechol groups for enhanced interfacial binding, antioxidant interfacial proteins (i.e., mfp-6) and hydrophobic interfacial protein (i.e., mfp-3s) were reported to be significant role. The reducing capacity of Mfp-6 is attributed to its side chain (i.e., cysteine residues, DOPA). It is reported that Mfp-6 has a reducing capacity of ~17 e^2212^ per protein; half of them contributed by cysteine residues, whereas the other half likely comes from DOPA [99]. Similarly, Dopa in Mfp-3 and Mfp-5 also being rescued by oxidation of thiol in cysteine residues for preserving its reduced form. Mfp-3s has demonstrated a lower loss of H-bonding interactions between its Dopa residues and mica substrate due to its hydrophobic property that can protect DOPA being autoxidation in basic pH [100].

Ionic species is another factor that can affect the adhesion between catechol group and substrate. Incorporating anionic functional group in containing catechol residue do not enhance the interfacial binding. The incorporation of cationic functional group to catechol groups containing adhesion elevate the interfacial binding to various inorganic substrate [101,102,103]. Narkar et al. [104] have made a systematic study about the effect of ionic side chain on oxidation state and interfacial binding property of catechol-functionalized hydrogel. They prepared adhesive hydrogels by copolymerizing dopamine methacrylamide (DMA) with either acrylic acid (AAc) or *N*-(3-aminopropyl)methacrylamide hydrochloride (APMH). The results showed that the ionic groups adhered to opposite charge surface via electrostatic interaction, and its adhesion values in some cases were higher than those of catechol. Weak oxidation of catechol groups was observed at a pH of 8.5 when incorporating the anionic groups (AAc), and correspondingly preserved the enhanced adhesive property of catechol to both quartz and amine-functionalized surfaces. Increasing pH to 9.0, catechol groups was oxidized and resulting lost buffering capacity of AAc. On the other hand, cohesive covalent bond was formed at a basic pH when incorporated APMH with amine side chain into hydrogel, which interfered the interfacial binding capability of hydrogel and the substrate.

### 5.2. Effect of pH on Hydrogel Adhesion

Catechol groups can form stable, reversible, and pH-dependent complexes with metal ions and metal oxides. However, pH has different effects on the adhesion of catechol group to biological substrates. A catechol group can form irreversible covalent bonds with nucleophilic groups (e.g., -NH_2_ and -SH) in biological substrates through oxidation, thereby achieving adhesion. Cross-linking of these catechol-based hydrogels to biological substrates is affected by pH, thus influencing the curing rate and adhesive properties of catechol-functionalized hydrogels. Cencer et al. [105] investigated the effect of pH on the intermolecular cross-linking rate and adhesion to biological substrates using 4-armed PEG end-capped with DOPA (PEG-D) as a model adhesive polymer. They found reduced curing rate, mechanical properties, and adhesive performance to pericardium tissues at an acidic pH (5.7–6.7). Although a faster curing rate was observed at pH 8, the mechanical and bioadhesive properties of these adhesives were reduced compared to those buffered at pH 7.4. Adhesives formulated at pH 7.4 demonstrated a good balance of fast curing rate, improved mechanical properties, and interfacial binding. They concluded that stability of the transient oxidation intermediate of catechol groups was increased under acidic conditions, which reduced the intermolecular cross-linking rate and bulk adhesive properties of the hydrogels. At pH 8, the competitive cross-linking reaction mechanisms and reduced concentration of catechol groups due to auto-oxidation reduced the degree of polymerization and adhesive strength of these hydrogels.

The increase in pH contributes to the possibility of coordinate binding of DOPA to the oxide surface and increases the rate of DOPA oxidation, but it also reduces the number of DOPA residues available for binding. Yu et al. [97] examined the force-distance distribution and adhesion energy of Mefp-3 on TiO_2_ surface at three different pH values of 3, 5.5, and 7.5. They found the strongest adhesion force of Mefp-3 to TiO_2_ at pH 3, with an adhesion energy of ∼−7.0 mJ/m^2^, and a higher adhesion force at pH 7.5 than at pH. 5.5.

### 5.3. Effect of Molecular Weight on Hydrogel Adhesive Properties

Macroscopically, molecular weight affects the strength, viscosity, and toughness of polymer materials, all of which generally increase with higher molecular weight [106,107]. Microscopically, molecular weight affects the entanglement, interdiffusion, and interfacial interactions of molecular chains, all of which influence the adhesive properties of polymer materials [108]. The adhesive properties of catechol-functionalized polymer materials to substrates are also affected by a molecular weight. Lower molecular weight polymers, even monomers and oligomers, can interact more with the surface due to high mobility, and the resulting wetness provides many points of contact [108,109]. However, once the polymers adhere to the substrate surface, high molecular weight polymers provide a stronger surface force and are more efficient in dissipating energy when stressed compared to low molecular weight polymers [108].

Jenkins et al. [15] mimicked MAPs adhesion with synthetic poly[(3,4-dihydroxystyrene)-co-styrene] to understand how bulk adhesion of a mussel mimetic polymer varies with molecular weight. Their systematic structure-function studies were performed with and without an oxidative cross-linker. They concluded that molecular weight had a large effect on the bulk adhesion of the mussel mimetic DOPA-containing protein polymer system. Stronger adhesion was generally found at higher molecular weights without cross-linking. When a [N(C_4_H_9_)_4_](IO_4_) cross-linker was added, adhesion peaked at molecular weights of ∼50,000–65,000 g/mol. These data illustrate how changes in cohesion-adhesion balance affect bulk bonding. Considering that mussel adhesive plaques achieve this balance by incorporating several proteins with molecular weights ranging from 6000 to 110,000 g/mol, they mixed polymers with a range of molecular weights to mimic these different proteins. They found that this mixture had stronger adhesion than any individual polymer when cross-linked with the oxidant [N(C_4_H_9_)_4_](IO_4_).

## 6. Medical Applications of Catechol-Functionalized Hydrogels

### 6.1. Tissue Adhesion, Hemostasis, and Healing

To develop adhesives capable to improve cell attachment, proliferation and adhesion to tissues has been a challenge in biomedical applications. Inspiring from MAPs, catechol-functionalized hydrogels apply in medicine widely (e.g., tissue adhesion, hemostasis and healing) due to the adhesion in wet environment and well biocompatibility for tissues. Medical soft tissue adhesives are preparations, materials, or substances that are mainly applied for topical adhesion and repair of organs or tissues, supporting hemostasis with conventional sutures, and other technical fields because of their ability to adhere to soft tissues or provide inter-surface adhesion. Compared with conventional techniques such as suturing and stapling, medical adhesives can effectively shorten operation time and reduce pain, making them popular among doctors and patients. Ideal medical adhesives should be biodegradable, non-persistent, safe, non-toxic, non-carcinogenic, non-teratogenic, non-mutagenic, and biocompatible. Inspired by MAPs, natural or synthetic polymers are functionalized with catechol groups to prepare soft tissue adhesives that can adhere in a wet environment. To address the challenge of wet tissue adhesion, Mehdizadeh et al. [110] prepared an injectable citrate-based mussel-inspired bioadhesive (iCMBA) through a one-step reaction of citric acid, PEG and DOPA. The adhesive strength of hydrogels to porcine skin and acellular small intestine submucosa was determined by lap shear strength test. The results showed that these materials demonstrated rubber-like behavior with typical stress-strain curves of elastomers, which is especially important for soft tissue adhesives, and the bioadhesive offered 2.5–8.0-fold stronger wet tissue adhesion over the clinically used fibrin glue. Yet in clinical practice, the value of adhesive would be smaller than the measured, due to the distinct application conditions. Biocompatibility tests using NIH 3T3 fibroblast cells by MTT assay has showed the compatibility of cyto/tissue in vitro; and in vivo study, wound healing properties were tested using rat skin incision model, the results confirmed the effectiveness and convenience of using iCMBAa in wound closure. Moreover, it was able to stop bleeding instantly without sutures and could be completely degraded and absorbed without causing a significant inflammatory response. Therefore, it can be used as a surgical tissue adhesive, sealant, and hemostatic agent.

In order to improve the adhesive strength, which is the main index of tissue adhesives, Chen et al. [96] reported a novel mussel-inspired tissue-adhesive hydrogel consisting of poly(γ-glutamic acid) and DOPA (γ-PGA-DA) for tissue adhesion as well as hemostasis. They demonstrated that the prepared hydrogel could bond tissues well and stop bleeding in a wet environment via a horseradish peroxidase-mediated reaction. The hydrogel exhibited 10–12-fold stronger wet tissue adhesion strength (58.2 kPa) over clinically used fibrin glue, and provided more effective hemostasis after liver puncture in animal models (41.2% less bleeding compared with fibrin glue). Moreover, the hydrogel showed superior cyto/tissue compatibility and demonstrated controlled gelation time, swelling ratio, microscopic morphology, biodegradability, tissue-like elastomeric mechanical properties. Overall, the γ-PGA-DA hydrogel was shown to be a promising wet-resistant adhesive and hemostatic.

At present, available adhesives require non-toxic, biocompatibility and biodegradability. Compared with the commonly used adhesives, however, these materials often have lower wet adhesive strength, longer bonding time and higher cost without modified. Natural polymer modified catechol groups may enhance the wet adhesive strength and potentially apply in clinical practice. The catechol-functionalized hydrogel studied by a large number of researchers have made the well wet adhesive strength, however, there need to be a long way to apply in clinical due to uncontrollable oxidation of catechol groups in weak acidic environment. In addition, the catechol-functionalized hydrogels often do not have the functions of wet adhesion, hemostasis and healing. In scientific research, most of the models are rabbits or rats, which is distinct from human physiological environment. Therefore, more clinical research should be taken on adhesive strength, hemostasis and healing, and more research need to be carried out on toxic and cell biocompatibility of degradation of hydrogels.

### 6.2. Antimicrobial and Anti-Infective Applications

**Figure 12:**
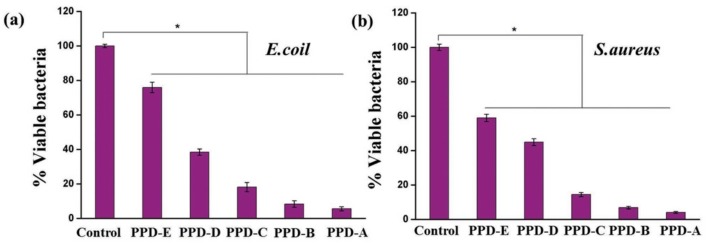
Antimicrobial properties of polyethylene glycol (PEG)-based hydrogels (PPD) hydrogels against various microbes. The bactericidal activity of PPD hydrogels with different DS of catechol (PPD-A: 2%, PPD-B: 5%, PPD-C: 8%, PPD-D: 18%, PPD-E: 25%) against (**a**) E. coli (Gram-negative) and (**b**) S. aureus (Gram-positive) (*n* = 3, * *p* < 0.05). Reproduced with permission from Ref. [115], Copyright 2017 Wiley-VCH Verlag GmbH & Co. KGaA.

The high-water contents of the aqueous physiological environment and hydrogels greatly diminish binding to the target tissue and can easily cause wound infections, thereby limiting the effectiveness of wound care management. The tight assembly of hydrogels and tissues and prevention of wound infections remain a major challenge. Wang et al. [111] prepared DOPA-modified ε-poly-L-lysine (EPL)-PEG-based hydrogels (PPD hydrogels) as wound dressings by cross-linking with horseradish peroxidase. The synergistic effect of catechol-Lys imparted excellent wet tissue adhesion properties to the hydrogels, thereby enabling easy, tight binding to biological tissue, producing effective hemostasis in vivo, and accelerating wound repair. As shown in Figure 12, the antimicrobial properties of the PPD hydrogels against *Escherichia coil* (Figure 12a) and *Staphylococcus aureus* (Figure 12b) decreased as the degree of containing catechol groups substitution increased. This trend was different from that of the adhesion strength, which they hypothesized was related to the free amino group content of the EPL backbone.

Amato et al. [112] prepared catechol-functionalized chitosan and studied the effects of different catechol groups levels on the inherent antimicrobial activity of the polymer itself. They found enhanced antimicrobial activity of chitosan after catechol groups functionalization, with a four-fold reduction in the polymer minimum inhibitory concentration over S. epidermidis. Guo et al. [113,114] introduced the clinically used and inexpensive anti-fungal agent, 10-undecylenic acid, into their previously developed iCMBAs and used sodium metaperiodate as a cross-linker to develop a new type of hydrogel, called AbAf iCs. AbAf iCs demonstrated strong wet tissue adhesion strength and also exhibited excellent in vitro cyto compatibility, rapid degradation, and strong initial and considerable long-term antimicrobial and antifungal activities. Compared with the control, experimental groups have stronger antimicrobial activities lasting at least 24h against S.aureus and E. coli. The fungal inhibition evaluation suggested that the tested AbAfiCs cross-linked by either SN or PI both exhibited even better antifungal performance against C.albicans than their antibacterial performance. The authors suggested that AbAf iCs could serve as excellent antibacterial and antifungal bioadhesive candidates for tissue/wound closure, wound dressing, and bone regeneration, especially when bacterial or fungal infections are a major concern. 

It is generally known that reactive oxygen species such as super oxide anion and hydrogen peroxide are generated during catechol groups oxidation [115]. Hydrogen peroxide is a widely used disinfectant in medicine but it is not easy to store and transport due to its oxidant. Because hydrogen peroxide is generated during the oxidation of catechol groups, catechol-functionalized microgels have been prepared by Meng et al. [116]. They found that the microgels reduced the infectivity of the more biocide resistant non-envelope virus by 3 log reduction value (99.9% reduction in infectivity) and microgels can be repeatedly activated and deactivated for hydrogen peroxide generation by controlling the oxidation state of catechol groups. These microgels did not contain a reservoir for storing the reactive hydrogen peroxide and could potentially function as a lightweight and portable dried powder source for the disinfectant for a wide range of applications in medicine.

Antimicrobial catechol-functionalized hydrogel has a wide application prospect in the field of medicine. Especially, as a tissue adhesive, it can not only effectively adhere to tissue, but also inhibit the growth of bacteria, and thus promoting the tissue rapid healing. This will bring great convenience in clinic. On the other hand, the main models investigated on antibacterial assay are Escherichia coli, Staphylococcus aureus and Staphylococcus epidermidis, which shown the well antimicrobial properties, however, in clinical practice, tissue infection is not only the representative strains mentioned above, there may be a lack of research on other strains. In addition, the ideal antimicrobial agent is expected to last for a long time which is conducive to cell proliferation and repair. Most studies have proved that the antimicrobial catechol-functionalized hydrogel has antimicrobial properties, but rarely involves long-term antimicrobial agents. In fact, catechol moieties could have an intrinsic antimicrobial activity, as reported for some catechol bearing natural extracts [117], but the mechanism is still unclear.

### 6.3. Biomedical Coatings

#### 6.3.1. Bone Regeneration and Orthopedic Implant Coatings

**Figure 13:**
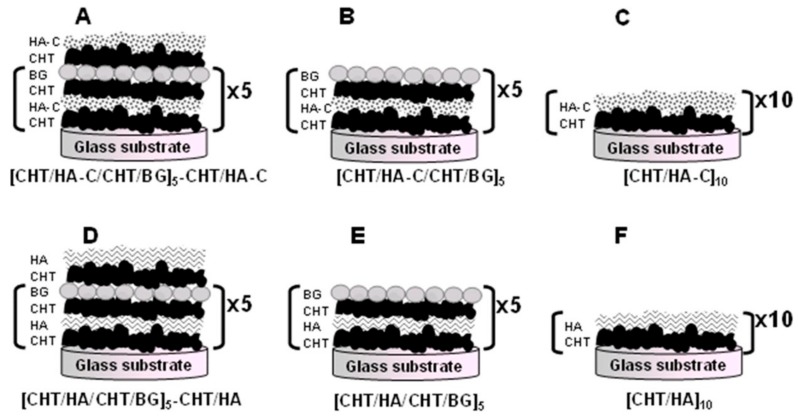
Schematic of the multilayer film configuration of (**A**) condition 1, [CHT/HA-C/CHT/BG]_5_-CHT/HA-C; (**B**) condition 2, [CHT/HA-C/CHT/BG]_5_; (**C**) condition 3, [CHT/HA-C]_10_; (**D**) control 1, [CHT/HA/CHT/BG]_5_-CHT/HA; (**E**) control 2, [CHT/HA/CHT/BG]_5_; and (**F**) control 3, [CHT/HA]_10_. Reproduced with permission from Ref. [128], Copyright 2015, American Chemical Society.

**Figure 14:**
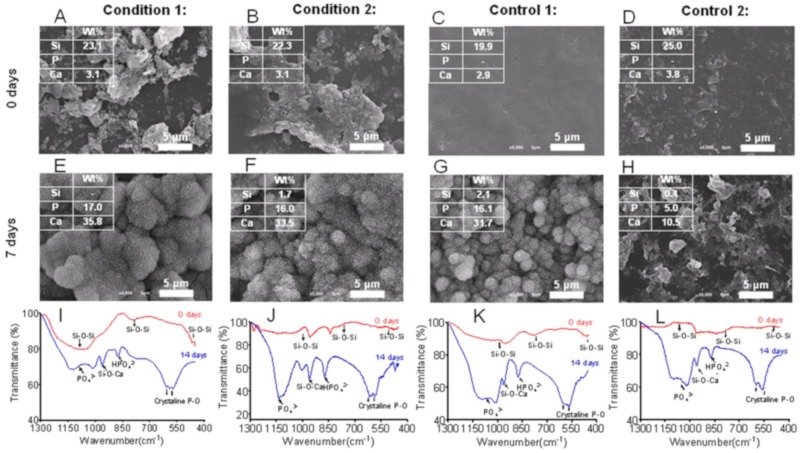
In vitro bioactivity studies. (**A**–**H**) Representative images of scanning electron microscopy with quantitative EDS analysis of four LBL film configurations (condition 1, [CHT/HA-C/CHT/BG]_5_-CHT/HA-C; condition 2, [CHT/HA-C/CHT/BG]_5_; control 1, [CHT/HA/CHT/BG]_5_-CHT/HA; and control 2, [CHT/HA/CHT/BG]_5_) (**A**–**D**) before and (**E**–**H**) after the immersion in simulated body fluid (SBF) for 7 days. (**I**–**L**) Corresponding FTIR spectra obtained before and after the immersion in SBF for 14 days. Reproduced with permission from Ref. [128], Copyright 2015, American Chemical Society.

It is generally recognized that some ceramics and glasses can bind to bone without forming a fibrous capsule [118,119]. When in contact with bone, the implanted bioactive material chemically bonds to the bone by forming a hydroxyapatite layer that is identical to the bone mineral [119]. Bioactive glass is the most famous example. In addition to being biologically active, bioactive glass nanoparticles can improve osteoconductivity, cell proliferation, differentiation, and vascularization [120,121,122,123]. Inspired by MAPs, Rego et al. [124] developed organic-inorganic multilayered films based on bioactive glass nanoparticles (BG), chitosan (CHT), and hyaluronic acid modified with catechol groups (HA–C). As Figure 13 shown, different samples have been prepared by layer-by-layer (LBL) method. The adhesion tests results shown that [CHT/HA-C/CHT/BG]+CHT/HA-C films which containing catechol groups on outer layer present an adhesive strength of 2.09 ± 0.04 MPa, whereas the value for [CHT/HA-C/CHT/BG] which contains bioglass nanoparticles on its outer layer is 1.16 ± 0.04 MPa. Moreover, with or without nanoparticles in their constitution, multifunctional films containing hyaluronic acid modified with dopamine demonstrated enhanced adhesive properties. Apatite formation was investigated under physiological-like conditions onto the films by in vitro bioactivity tests. Before the simulated body fluid (SBF) immersion (0 days) (Figure 14), it is possible to observe heterogeneous nanoparticles agglomerations on the film surface for all the film configurations. After immersion (7 days) (Figure 14), the SEM images evidenced that the surface of coatings presents apatite-like structures with the typical cauliflower morphology. The results confirmed the formation of apatite. It also demonstrated that the samples without bioglass nanoparticles do not present the bioactive character. The result of Fourier transform infrared has also confirmed the conclusion (Figure 14). In vitro biocompatibility has been invested using L929 cells, and demonstrated the well proliferation and biocompatibility. With enhanced adhesion and bioactivity, the mussel-inspired multi-functional multilayered films can potentially be used as coatings for orthopedic implants.

Zhang et al. [125] immobilized mouse bone marrow stem cells in a hydrogel composed of alginate, alginate-DOPA beads, and fibers to study its biological interactions as a bone regeneration coating. Dopamine-modified alginates promoted cell survival and proliferation, and the alginate-DOPA gel promoted osteogenic differentiation of mesenchymal stem cells. Moreover, the adhesive properties of DOPA allowed for coating the surface of the alginate-DOPA gel with silver nanoparticles, thereby imparting significant antimicrobial activity. They therefore concluded that DOPA-modified alginate gel could be used for cell encapsulation to promote cell proliferation and could be applied to bone regeneration, especially in bone defects. Another work of Zhang et al. [126] showed multilayer films of dopamine-modified hyaluronic acid/chitosan (DHA/CHI) built on the surface of Ti-24Nb-2Zr (TNZ) alloy can improve osteoblast (MC3T3-E1) proliferation and biocompatibility for orthopedics applications when compared with to original ones.

Utilizing the wet adhesive property of catechol groups, the catechol-functionalized hydrogels coating can not only enhance the adhesive strength to various surface of substrate (e.g., titanium alloy) that potentially applying in bone regeneration and orthopedic implant, but also improve cell proliferation during the clinical process. However, further study should be taken for overcome the obstacle of weak adhesive strength with various pH in physiological environment.

#### 6.3.2. Heparin-Mimetic Coatings

Heparin has long been used as an anticoagulant in clinical practice. It is also widely employed for the surface modification of blood-contacting and biomedical materials [127,128,129]. Heparin-mimetic anticoagulant molecules have also been attached to the surfaces of biological materials [130,131,132]. To obtain stable surface-modified coatings, a range of anticoagulant coatings of artificial biomaterials, such as hydrogel coatings, have been designed and assessed [133,134,135]. Ma et al. [136] synthesized sodium alginate (SASS) with different degrees of sulfation, yielding a chemical structure and bioactivity similar to heparin. The DOPA was grafted onto the SASS surface to obtain surface-modified DA-g-SASS, which was then coated onto the polymer surface by the adhesion of catechol groups. The heparin-mimetic coatings significantly promoted cell adhesion and proliferation and inhibited the thrombotic potential and inflammation induced by the material interface. The authors suggested that heparin-mimetic coatings could be used for surface anticoagulant modification of various biological and clinical devices for blood purification, tissue implantation, and other micro-/nanomaterials.

Heparin has potential applications for enhancing the blood compatibility of various biomedical devices such as catheters, grafts and stents. Existing approaches to heparin immobilization are based on covalent bond formation and electrostatic interactions between substrates and heparin molecules. However, these approaches are limited by complex multi-step procedures and uncontrolled desorption of heparin. You et al. [137] prepared a highly surface-adhesive heparin derivative by attaching the mussel-inspired adhesive molecule DOPA to the heparin backbone. Immersion of poly(urethane) substrates into an aqueous solution of the heparin derivative resulted in a strong heparin coating of the poly(urethane), the most widely used polymer material for blood-contacting medical devices. The heparin-DOPA-modified derivative-coated poly(urethane) substrate significantly inhibited blood coagulation and platelet adhesion.

#### 6.3.3. Antimicrobial Coatings

Unlike Fullennkamp et al. [19], who developed nano-silver as an antimicrobial adhesive via oxidation of catechol groups by silver ions, García-Fernández et al. [138] introduced chlorine into the benzene ring of catechol to prevent bacterial biofilm formation. In their work, 2-chloro-4,5-dihydroxyphenylalanine (Cl-DOPA) was grafted to the end of PEG and mixed with the polymer at the end of the DOPA-grafted PEG in certain ratios, thereby forming hydrogels based on DOPA reactivity. The prepared hydrogels were able to prevent bacterial adhesion due to the presence of the Cl-DOPA group and had no toxic effects on the attached cells. This Cl-DOPA-functionalized biomaterial can be widely used in antimicrobial coatings for a variety of biomedical devices.

### 6.4. Drug Delivery

Hydrogel polymer networks are filled with a large amount of water that impart the characteristic of soft, wet surfaces and affinity with tissues to it. These properties greatly reduce the stimulation of materials to surrounding tissues, making hydrogels possess potential value in drug delivery systems. However, hydrogels are easily damaged, which limit their application in drug delivery system. Therefore, improving the mechanical strength of hydrogels in swelling state is concerned by many researchers. Catechol-functionalized hydrogels are payed attention for its wet adhesive properties and biocompatibility. Numerous adhesive polymers have been developed to increase the residence time of formulated drugs at specific sites. However, it has been difficult to obtain satisfactory adhesive properties. Although thiolation and lectin functionalization have been extensively studied, the reversibility of disulfide bonds in the case of vulcanization and lectin toxicity remains a challenge. Inspired by MAPs, Kim et al. [22] prepared an adhesive for drug delivery by grafting adhesive moiety with catechol groups (i.e., 3,4—dihydroxy hydrocinnamic acid) to the well-known polymer that is chitosan. Gastrointestinal tract retention of the modified chitosan was improved compared to unmodified chitosan due to the irreversible catechol-mediated cross-linking of catechol groups with mucin. This formulation may represent a new generation of adhesive polymers in mucosal drug delivery.

Given the great demand for new treatments for Parkinson’s disease, DOPA-based injectable polysaccharide hydrogels have great potential as a local drug delivery system. Ren et al. [24] prepared a DOPA-based and poly-DOPA cross-linked injectable hydrogel using a mixture of oxidized quaternized chitosan, gelatin, and DOPA. DOPA (as a drug for treating Parkinson’s disease) and metronidazole (as an anti-inflammatory drug) were encapsulated in the hydrogel. The release profiles indicated that the injectable hydrogel had a large capacity as a carrier for the long-term local release system of DOPA and metronidazole. Moreover, the cytocompatibility of the hydrogel was confirmed by cell viability and proliferation assays using mouse L929 fibroblasts. Therefore, this material can be used as a long-term, injectable, sustained release system for DOPA and an anti-inflammatory drug to treat Parkinson’s disease. Catechol-functionalized hydrogel was studied on drug delivery seem to encouraging. However, how to control drug release from hydrogels needs further study.

### 6.5. Tissue Engineering Scaffolds

Tissue engineering scaffold materials are functional materials specifically developed for the tissue to be replaced; they can bind to living tissue cells and be implanted in different tissues. Cell scaffolds composed of biological materials, which serve as artificial extracellular matrices, are required for the proliferation and differentiation of seed cells. Hydrogels are hydrophilic polymers swollen by large amounts of water and are widely used in biomedical applications for tissue engineering. Their high water content and soft porous 3D structure mimics the in vivo extracellular matrix (ECM) microenvironment making them useful for biomedical applications. Lee et al. [139] prepared a hydrogel by grafting DOPA onto alginate backbones via oxidative cross-linking to develop an adjustable, functional, biocompatible, 3D scaffold for tissue engineering and cell therapy. Oxidative cross-linking of catechol groups instead of divalent cations reduced cytotoxicity and enhanced the survival of various human primary cells (e.g., stem cells) in a 3D gel matrix. Moreover, the in vivo inflammatory response was significantly reduced compared to that induced by conventional alginate hydrogels with calcium cross-links. The characteristic of natural polymer materials for tissue engineering scaffolds is that their degradation products are easily absorbed by the body, but their strength and processing performance are poor, and the degradation rate cannot be adjusted. Adding materials to hydrogels or grafting with functional groups such as catechol groups on natural polymer may enhance the mechanical or adhesive properties. Catechol-functionalized hydrogels, with wet adhesive properties and well compatibility with cells, may have potential value for tissue engineering scaffold on which should be more concern.

### 6.6. Islet Transplantation

Islet transplantation has been widely studied as a potential cure for diabetes [140]. In experimental diabetic animals, islet transplantation can correct metabolic abnormalities, stabilize blood glucose, relieve hypoglycemic episodes, and prevent the development and progression of diabetic microangiopathy [141]. However, inflammation insult, may result to unsuccessful transplantation, would be inevitable emerged during the islet transplantation. As a proven ideal treatment of insulin-dependent diabetes, islet transplantation can be improved by the use of catechol-functionalized hydrogels due to stronger adhesion to wet tissue surfaces. Brubaker et al. [142] directly attached islets to the tissue surface using catechol-functionalized branched PEG as an “islet sealant,” thus avoiding functional impairment associated with intrahepatic portal vein infusion. The catechol-functionalized hydrogel induced minimal or acute inflammatory responses following islet transplantation in C57BL6 mice and maintained an intact tissue interface for up to one year. It also provided effective immobilization transplanted islets at the epididymal fat pad and external liver surfaces, thereby allowing glucose recovery and revascularization. Islet sealant could attach islets to the tissue surface that would favor for the trouble of requiring great quantities islets during transplantation. Similar to other medical applications, the adhesive strength should be enhanced and need further studies on clinical practice.

## 7. Conclusions and Prospects

MAP adhesion to different substrate surfaces is due to the redox of DOPA and chelation with different metals, as well as intermolecular chemical interactions between other amino acid residues (e.g., charged, hydrophobic, and antioxidant thiol residues) and multiple Mfps. Mussels also minimize structural damage associated with contact deformation between two different materials (i.e., biological tissue and mineralized surface) based on optimized structural and geometric design by classifying different proteins by performance. All these factors need to be considered when designing catechol-functionalized hydrogels.

The binding of catechol groups to organic interfaces mainly depends on oxidation to form quinone, which is then involved in intermolecular covalent cross-linking. However, this oxidation occurs on the basis of rapid consumption of catechol groups, inevitably reducing its levels, which may decrease the adhesive strength of the catechol-functionalized hydrogels. On the other hand, catechol-functionalized hydrogels which experience the oxidation in alkaline environment would have the elevated adhesion to tissue substrate. However, there would be weaker adhesion in mildly acidic environment (e.g., cancer cell (pH ≤ 7) [143], skin (pH 4–6) [144], and subcutaneous tissues (pH 6.7–7.1) [145]). This would be an obstacles or challenges on clinical applications. Through the adhesion of biomimetic MAPs, if we can find a protein (or amino acid) that can adhere to biological tissues under acidic conditions, it will open the way for the clinical application for catechol-functionalized hydrogels. Moreover, catechol groups produce active oxygen during oxidation. The production of active oxygen is related to the degree of oxidation. A moderate level of active oxygen is conducive to sterilization and disinfection, whereas excessive reactive oxygen can cause inflammation. This needs to be considered when preparing catechol-functionalized hydrogels.

Despite many obstacles, there have been continuous efforts to study and develop catechol-functionalized hydrogels, which are a promising material in a range of medical applications. They provide effective adhesion at the wet tissue interface, offering obvious advantages in adhesion, hemostasis, and healing of wounded tissue. Optimized hydrogels can also be used as coatings for biological and clinical devices, carriers for drug delivery, and tissue engineering scaffolds. With continued research efforts, catechol-functionalized hydrogels will undoubtedly play important roles in the medical field and have wider applications.

## Figures and Tables

**Table 1:** Adhesion strength of double cross-linked tissue adhesive hydrogel (DCTA) under different conditions. Reproduced with permission from Ref. [72], Copyright 2016, Acta Materialia Inc.

Adhesion Pattern	Adhesion Strength (kPa)
Rapid-curing skin-fat gluing	9.3 ± 4.9
2-h-curing skin-fat gluing	24.7 ± 3.3
24-h-curing skin-fat gluing	12.9 ± 0.5
2-h-curing skin-collagen gluing	20.4 ± 4.0
2-h-curing cartilage gluing	194.4 ± 20.7

**Table 2:** Information of MAPs, recombinant MAPs and catechol-functionalized materials on different substrates.

Substrate	Materials Used	Crosslinker	Reported Adhesive Strength	References
Mouse Subcutaneous Tissue	^a^ CHI-C/Plu-SH	None	15.0 ± 3.5 kPa	[67]
Mouse Subcutaneous Tissue	CHI/Pluronic F127	None	1.9 ± 1.8 kPa	[67]
Mouse Subcutaneous Tissue	CHI/Plu-SH	None	5.3 ± 2.6 kPa	[67]
Mouse Subcutaneous Tissue	CHI-C/Pluronic F127	None	6.6 ± 1.0 kPa	[67]
Rabbit Small Intestinal Mucosa	Physical Mixture of Chitosan, Hydrocaffeic Acid	NaIO_4_	1.01 ± 0.09 kPa	[94]
Rabbit Small intestine	Chitosan	None	0.35 ± 0.05 kPa	[94]
Rabbit Small intestine	Physical Mixture of Chitosan, Hydrocaffeic Acid	None	0.76 ± 0.11 kPa	[94]
Porcine Skin	Recombinant MAP	NaIO_4_ and F^3+^	~200 kPa	[95]
Porcine Skin	^b^ γ-PGA-DA	H_2_O_2_ and HRP	58.2 kPa	[96]
Gelatin-coated Glass	^c^ PLGA/ALG-CHO	Schiff Based Reaction	8.5 ± 1.0 kPa	[72]
Gelatin-coated Glass	^d^ PLGA/ALG-CHO-Catechol	Hydrogen Bonding and Schiff Based reaction	24.9 kPa	[72]
Porcine Skin	PLGA/ALG-CHO-Catechol	Hydrogen Bonding and Schiff Based reaction	15.5 kPa	[72]
Wet Rat Skin	^e^ HCA-g-HECTS	NaIO_4_	73.56 kPa	[93]

^a^ CHI = chitosan, CHI-C/Plu-SH =catechol-functionalized chitosan/thiol-terminated pluronic F-127. ^b^ γ-PGA = Poly(γ-glutamic acid), DA = dopamine. ^c^ PLGA = adipic dihydrazide-modified ploy(L-glutamic acid), ALG-CHO = aldehyde-modified alginate. ^d^ ALG-CHO-Catechol = catechol- and aldehyde-modified alginate. ^e^ HCA = hydrocaffeic acid, HECTS = hydroxymethyl chitosan.
